# Effects of Prenatal Exposure to Polychlorinated Biphenyls and Dioxins on Mental and Motor Development in Japanese Children at 6 Months of Age

**DOI:** 10.1289/ehp.8614

**Published:** 2005-12-15

**Authors:** Sonomi Nakajima, Yasuaki Saijo, Shizue Kato, Seiko Sasaki, Akiko Uno, Nobuo Kanagami, Hironori Hirakawa, Tsuguhide Hori, Kazuhiro Tobiishi, Takashi Todaka, Yuji Nakamura, Satoko Yanagiya, Yasuhito Sengoku, Takao Iida, Fumihiro Sata, Reiko Kishi

**Affiliations:** 1 Department of Public Health, Hokkaido University Graduate School of Medicine, Sapporo, Japan; 2 Department of Occupational Therapy, School of Health Sciences, Sapporo Medical University, Sapporo, Japan; 3 Sapporo Toho Hospital, Sapporo, Japan; 4 Fukuoka Institute of Health and Environmental Sciences, Fukuoka, Japan; 5 Japan Food Hygiene Association, Tokyo, Japan; 6 Division of Sensory Integrative Dysfunction, Department of Occupational Therapy, Sapporo Medical University Graduate School of Health Sciences, Sapporo, Japan

**Keywords:** dioxins, infant development, maternal blood, polychlorinated biphenyls (PCBs), prenatal exposure

## Abstract

Several studies have shown that prenatal and/or postnatal background-level exposure to environmental chemicals, such as polychlorinated biphenyls (PCBs) and dioxins, induces adverse effects on the neurodevelopment of children. However, other studies have not detected any harmful influences on neurodevelopment. Furthermore, except in western countries, no developmental tests have been carried out in relation to detailed assessment of exposure to PCBs and dioxins. In this study (the Hokkaido Study on Environment and Children’s Health), the effect of prenatal exposure to background levels of PCBs and dioxins on infant neurodevelopment in Japan/Sapporo was elucidated. The associations between the total or individual isomer level of PCBs and dioxins in 134 Japanese pregnant women’s peripheral blood and the mental or motor development of their 6-month-old infants were evaluated using the second edition of the Bayley Scales of Infant Development. The mean level of total toxicity equivalency quantity (TEQ) was 18.8 (4.0–51.2) pg/g lipid in blood of 134 mothers. After adjustment for potential confounding variables, the total TEQ value was shown not to be significantly associated with mental developmental index (MDI) or psychomotor developmental index (PDI). However, the levels of one polychlorinated dibenzo-*p*-dioxin (PCDD) isomer, total PCDDs, and total PCDDs/polychlorinated dibenzofurans (PCDFs) were significantly negatively associated with MDI, and the levels of two PCDD isomers and three PCDF isomers were significantly negatively associated with the PDI. In conclusion, the background-level exposure of several isomers of dioxins during the prenatal period probably affects the motor development of 6-month-old infants more than it does their mental development.

Polychlorinated biphenyls (PCBs) and dioxins are persistent environmental pollutants that occur in the environment. They have varying influences on the human body. Some harmful influences of prenatal and/or postnatal exposure to PCBs and dioxins on the neurodevelopment of infants have previously been shown. Longitudinal studies have also been performed in both low-level (background-level) dioxin-contaminated areas and high-level contaminated areas to demonstrate the effects of these pollutants on the neurodevelopment of children.

Dozens of studies in background-level contaminated areas have been performed since the latter half of the 1980s, such as the North Carolina (USA) study (Gladen and Rogan 1991; Gladen et al. 1988, 2000; Rogan and Gladen 1991; Rogan et al. 1986), the Netherlands two-cities study (Huisman et al. 1995a, 1995b; Koopman-Esseboom et al. 1996; Lanting et al. 1998; Patandin et al. 1999; Vreugdenhil et al. 2002a, 2002b, 2004a, 2004b), the Düsseldorf, Germany, study (Walkowiak et al. 2001; Winneke et al. 1998), and the U.S. 11-cities study (Daniels et al. 2003; Gray et al. 2005). The Bayley Scales of Infant Development (BSID) or the BSID second edition (BSID-II) was used in all of these studies to evaluate infants’ mental or motor development. As a result, the prenatal and/or postnatal exposure was shown to have a significant negative association with motor development during infancy, whereas significant associations were not found in young children (Gladen and Rogan 1991; Gladen et al. 1988; Koopman-Esseboom et al. 1996; Patandin et al. 1999; Rogan and Gladen 1991; Vreugdenhil et al. 2002a, 2004a). On the other hand, only one study showed a significant negative association between postnatal exposure and the cognitive development of young children (Walkowiak et al. 2001). In addition, another study showed a positive association between prenatal exposure and motor development during infancy in some of the diverse study sites across the United States (Daniels et al. 2003).

Thus, consistent results relating to the influence of background-level dioxin exposure on children’s development have not been obtained. Longnecker et al. (2003) suggested that interpretation of human data regarding low-level, early-life PCB exposure and subsequent neurodevelopment was problematic because levels of exposure were not similarly quantified across studies.

In Japan, Tada and Nakamura (2000) examined the mental development of 1-year-old breast-fed infants and bottle-fed infants and reported that their mental development was within a normal range. However, this report was not based on detailed developmental tests such as BSID. Nagayama et al. (2004) examined the association between hexachloro-cyclohexane, heptachlor epoxide, chlordane, dieldrin, dichlorodiphenyltrichloroethane (DDT), PCBs, or dioxin in breast milk and the development of infants at 10 months. In that study, the authors evaluated the associations using the Enjohji Scales of Infant Development and reported that a significant negative association existed between PCBs and the development of comprehension. The developmental tests that were used in these two studies were different from those of previous studies, and it was not possible to perform detailed evaluations. An additional problem was that detailed exposure measurements had not been performed in these studies.

The aim of the present study was to examine the influence of environmental chemicals such as PCBs and dioxins on the mental and motor development of infants of the next generation in Sapporo, Japan. Consequently, we carried out detailed measurements of the levels of PCBs and dioxins in the mothers’ peripheral blood and performed neurodevelopmental assessments of their 6-month-old infants using BSID-II to examine the influence of prenatal exposure of these pollutants on the neurodevelopment of infants.

## Materials and Methods

### Study population.

We recruited pregnant women between July 2002 and July 2004 from the Sapporo Toho Hospital in Hokkaido, Japan (this study became known as the Hokkaido Study on Environment and Children’s Health). All the subjects were native Japanese and were resident in Sapporo and the surrounding areas. The subjects completed the self-administered questionnaire survey after the second trimester during their last pregnancy. The questionnaire provided information relating to their dietary habits, exposure to chemical compounds in their daily life and at their work site, home environment, smoking, and medical histories of themselves and their partners. The prenatal information of the mothers and their children was collected from their medical records. This study was conducted with all the subjects’ written informed consent and was approved by the institutional ethical board for epidemiologic studies at Hokkaido University Graduate School of Medicine.

### Exposure measures.

A 40-mL blood sample was taken from the maternal peripheral vein after the second trimester during their last pregnancy. When we were not able to take mother’s blood due to the mother’s anemia during pregnancy, we took the blood during hospitalization after delivery. All samples were stored at −80°C until analysis.

The concentrations of PCBs and dioxins in the maternal blood were measured using high-resolution gas chromatography/high-resolution mass spectrometry (HRGC/HRMS) equipped with a solvent-cut large-volume injection system (SGE Ltd., Victoria, Australia) at Fukuoka Institute of Health and Environmental Sciences. The gas chromatograph was an Agilent 6890 (Agilent Technologies Inc., Palo Alto, CA, USA) equipped with an AutoSpec-Ultima NT (Micromass Ltd., Manchester, UK). The levels of PCBs and dioxins were measured in each isomer [seven polychlorinated dibenzo-*p*-dioxins (PCDDs): 2,3,7,8-tetrachlorodibenzo-*p*-dioxin (TCDD), 1,2,3,7,8-pentachlorodibenzo-*p*-dioxin (PeCDD), 1,2,3,4,7,8-hexachlorodibenzo-*p*-dioxin (HxCDD), 1,2,3,6,7,8-HxCDD, 1,2,3,7,8,9-HxCDD, 1,2,3,4,6,7,8-hep-tachlorodibenzo-*p*-dioxin (HpCDD), octachlorodibenzo-*p*-dioxin (OCDD); 10 polychlorinated dibenzofurans (PCDFs): 2,3,7,8-tetrachlorodibenzofuran (TCDF), 2,3,7,8-pentachlorodibenzofuran (PeCDF), 2,3,4,7,8-PeCDF, 1,2,3,4,7,8-hexachloro-dibenzofuran (HxCDF), 1,2,3,6,7,8-HxCDF, 2,3,4,6,7,8-HxCDF, 1,2,3,7,8,9-HxCDF, 1,2,3,4,6,7,8-heptachlorodibenzofuran, 1,2,3,4,7,8,9-HpCDF, octachlorodibenzo-furan (OCDF); four non-*ortho* PCBs: 3,3′,4′,4′-tetracholorobiphenyl (TCB) (congener 77), 3,4,4′,5-TCB (congener 81), 3,3′,4,4′,5-pentachlorobiphenyl (PeCB) (congener 126), 3,3′,4,4′,5,5′-hexachlorobiphenyl (HxCB) (congener 169); eight mono-*ortho* PCBs: 2,3,3′4,4′-PeCB (congener 105), 2,3,4,4′,5-PeCB (congener 114), 2,3′,4,4′,5-PeCB (congener 118), 2′,3,4,4′,5-PeCB (congener 123), 2,3,3′,4,4′,5-HxCB (congener 156), 2,3,3′,4,4′,5′-HxCB (congener 157), 2,3′,4,4′5,5′-HxCB (congener 167), 2,3,3′,4,4′,5,5′-heptachlorobiphenyl (HpCB) (congener 189); and two di-*ortho* PCBs: 2,2′3,3′,4,4′,5-HpCB (congener 170), 2,2′,3,4,4′,5,5′-HpCB (congener 180)], and the total toxicity equivalency quantity (TEQ) levels were calculated (Iida and Todaka 2003; Todaka et al. 2003). Furthermore, for several subjects, 68 PCBs (including mono-*ortho* PCBs), which remained behind in the blood, were measured using HRGC/HRMS.

### Developmental measures.

We used BSID-II (Bayley 1993) to assess the infants’ mental and psychomotor development (mental, motor) at 6 months of age. BSID-II is a developmental test tool that is standardized for use in the United States and is most widely used as an infant assessment instrument in both clinical and research settings in the United States. The BSID-II mental scale assesses the age-appropriate children’s level of cognitive, language, and personal/social development. The motor scale assesses fine and gross motor development. Mental and motor scores are based on the calibration scale from raw score and are represented as index scores. The mean values of the mental developmental index (MDI) and the psychomotor developmental index (PDI) were 100, and the standard deviation was 15. Although BSID-II has a lot of strengths and weaknesses (Bayley 1993), it is a most useful test to measure the present attainment of developmental abilities of normal children (Bradley-Johnson 2001).

Because BSID-II was not standardized in Japan, we translated a BSID-II manual in consultation with a manual for BSID, which was used in the development study reported by Kato et al. (1987, 1988). The children were brought to the community center in Sapporo, where they were tested in a quiet, private room in the presence of the parent(s) by one examiner. The development evaluation was performed by three occupational therapists who have clinical experience in the field of developmental disabilities. The examiners were unaware of the infants’ PCB and dioxin exposure levels. First, for all examined children, the scoring was performed by the examiner who performed the examination, and then the scoring was double-checked by two other examiners based on a video that recorded the examination.

We investigated the environmental conditions of the subjects using the questionnaire of home environment devised by Anme et al. (1997).

### Data analysis.

We used the following eligibility criteria for analysis of subjects: no serious illnesses or complications during pregnancy and delivery, singleton babies born at term (37–42 weeks’ gestation), Apgar score of > 7 at 1 min, infants without congenital anomalies or diseases, and BSID-II completed.

We performed multiple-regression analysis to examine the association between BSID-II scores (MDI, PDI) and the levels of PCBs and dioxins in maternal blood. The levels of PCBs and dioxins in maternal blood were logarithmically transformed, and the analysis was adjusted for gestational age (days), smoking during pregnancy, and caffeine intake during pregnancy (milligrams per day). When we examined the levels of PCBs and dioxins among blood sampling time (during pregnancy and after delivery) by the Mann-Whitney test, there were significant differences in the levels of OCDD (*p* < 0.001), 1,2,3,4,6,7,8-HpCDD (*p* < 0.001), total PCDDs (*p* < 0.01), and total PCDDs/PCDFs (*p* < 0.05) (data not shown). So the blood sampling time was adjusted in multiple regression analysis. Results were considered significant if *p* < 0.05.

## Results

One hundred thirty-five mother–infant pairs fulfilled the determined eligibility criteria of this study; they completed exposure measurements and BSID-II. One pair was excluded from the study because the PCB and dioxin levels in the maternal blood were extremely high. So, in total, 134 mother–infant pairs were included in the study.

Characteristics of mothers and infants are presented in Table 1. The mean (± SD) maternal age was 31.1 ± 4.7 years; 13.4% of mothers continued smoking during pregnancy. Forty-four percent of mothers ate inshore fish during pregnancy at least once per week, and 58.2% of mothers ate deep-sea fish during pregnancy at least once per week. Fifty percent of infants were first-born, and 58.2% of infants had been breast-fed > 3 months. The mean scores of MDI and PDI were 91.9 ± 5.8 and 89.3 ± 10.5, respectively. Both values were lower than the standardized score.

The relationships between MDI and PDI scores and the subjects’ characteristics are presented in Table 2. For mothers’ characteristics, caffeine intake during pregnancy was significantly negatively associated with PDI scores (*r* = −0.177, *p* = 0.04). For infants’ characteristics, gestational age (days) was significantly positively associated with MDI scores (*r* = 0.178, *p* = 0.039) and PDI scores (*r* = 0.289, *p* = 0.001).

The levels of PCBs and dioxins (picogram per gram lipid) in maternal blood are presented in Table 3. For subjects with a level below the detection limit, we used a value equal to half the detection limit (Longnecker et al. 2000). The means (ranges) of levels of total PCDD/PCDF TEQ, total coplanar PCB TEQ, and total TEQ were 11.9 (2.1–31.2), 6.9 (1.1–22.2), and 18.8 (4.0–51.2) pg TEQ/g lipid, respectively. The mean level of dioxins in this study was slightly lower than that of the subjects in other studies of domestic areas with ages similar to that in this study. In addition, the median level of PCB-153 in the maternal blood of 64 subjects in this study was 22.9 ng/g lipid, which was lower than that in the previous study (Longnecker et al. 2003).

Table 4 shows the results of the multiple regression analysis of the association between the levels of PCBs and dioxins in maternal blood and MDI and PDI scores. After adjustment for blood sampling time, gestational age (days), smoking during pregnancy, and caffeine intake during pregnancy (milligrams per day), PCDD isomer 1,2,3,4,6,7,8-HpCDD (*p* < 0.05), total PCDDs (*p* < 0.01), and total PCDDs/PCDFs (*p* < 0.05) were significantly negatively associated with MDI. On the other hand, PCDD isomers 1,2,3,7,8,9-HxCDD (*p* < 0.05) and 1,2,3,4,6,7,8-HpCDD (*p* < 0.01), 2,3,7,8-TCDF (*p* < 0.05), 1,2,3,7,8-PeCDF (*p* < 0.05), and PCDF isomer 1,2,3,6,7,8-HxCDF (*p* < 0.05) were significantly negatively associated with PDI. The total levels of PCBs and dioxins were not significantly associated with PDI, and the TEQ values were not significantly associated with MDI or PDI.

## Discussion

To the best of our knowledge, this is the first report to investigate the association between the early neurodevelopment of infants and the total level and individual isomer level of Japanese pregnant women’s blood at background levels. As a result, there was no association between the TEQ value of the maternal blood and the PCB/dioxin level, whereas the levels of several isomers of dioxins were significantly negatively associated with mental or motor development. The total levels of PCDDs or PCDDs/PCDFs were significantly associated with mental development, whereas the levels of several specific isomers of PCDDs or PCDFs were associated with motor development.

In this study, the results of BSID-II were used as a neurodevelopment evaluation test for infants. The mean (± SD) MDI and PDI scores were 91.9 ± 5.8 and PDI 89.3 ± 10.5, respectively. Both scores were lower than the standardized scores (100). Because there is a cultural difference and a verbal difference between Japan and the United States, the BSID-II must be used with care in Japan. In particular, we should use standardized “classification” to judge how to treat or intervene with a child. However, the first BSID edition was used in Japan for developmental evaluation of children. In addition, Oka et al. (2005) reported that there was a high correlation between BSID-II and the Kyoto Developmental Test that was standardized in Japan. A study in Taiwan stated that reproducibility was high in BSID-II, even if there were cultural differences (Huang et al. 2000). Evaluation of neurodevelopment was limited to 6-month-old infants, and all the examiners scored all the infant subjects. Therefore, the BSID-II scores of the subjects in this study were directly comparable with each other.

The mean (range) level of PCBs and dioxins in maternal blood was 18.8 (4.0–51.2) pg TEQ/g lipid in this study, lower than that of subjects in studies in other domestic areas (Watanabe 2000). Longnecker et al. (2003) reported that interpretation of human data regarding low-level, early-life PCB exposure and subsequent neurodevelopment was problematic because levels of exposure were not similarly quantified across studies. Therefore, the PCB levels across studies of neurodevelopment were expressed in a uniform manner using the median level of PCB-153 in maternal pregnancy serum. The median PCB-153 level in the U.S. 11-cities, the Düsseldorf, the Netherlands two-cities, and the North Carolina studies ranged from 80 to 140 ng/g lipid, which were within the range of background levels. The median level of PCB-153 in the maternal blood of 64 subjects in this study was 22.9 ng/g lipid, which was clearly a lower contamination level in comparison with the other studies.

In this study, there was no significant association between the TEQ values of maternal blood PCB/dioxin level and mental and motor development, whereas there were significant negative associations with the levels of some isomers of dioxins and mental and motor development. The total levels of PCDDs or PCDDs/PCDFs were significantly associated with mental development, whereas the levels of several specific isomers of PCDDs or PCDFs were associated with motor development. There are many previous studies (Gladen et al. 1988; Koopman-Esseboom et al. 1996; Walkowiak et al. 2001) that show negative influences on the motor development of infants and young children rather than on their mental development. Although we used only the total PCB level or TEQ value as the exposure index in previous studies, we measured various levels of isomers of PCBs or dioxins in this study. In this study, there were no significant negative associations between the total levels of PCBs and dioxins and mental or motor development, whereas there were significant negative associations between motor development and levels of isomers of PCDDs and PCDFs. In this respect, it was impossible to explain the mechanism of our findings because there were few human or animal experimental studies investigating the association between individual isomer levels of PCBs and dioxins and neurodevelopment. So, at low-level exposure, total, and total TEQ values had only a minimal effect on mental and motor development. However, we found that several specific chemical compounds would have an adverse influence on motor development, whereas total PCDDs or total PCDDs/PCDFs would have an adverse influence on mental development.

Gray et al. (2005) reported that the level of total PCBs in mothers in their study, which did not show a significantly negative association with the level of total PCBs and the outcome, was similar to those in other studies in which an adverse effect was found. On the other hand, the mixture of PCBs in their specimens was unusual compared with that in other studies. Levels of nonquantitated PCBs may also have varied in biologically significant ways. Thus, they speculated that they might have had a relatively benign mixture if the composition of PCBs affected neurotoxicity. Therefore, the minimal effect of total and total TEQ values on mental and motor development in this study might be caused by the composition of PCBs and dioxins.

In this study, we performed multiple-regression analysis to examine the association between BSID-II scores (MDI, PDI) and total and individual isomer level of PCBs and dioxins. Because we performed 45 statistical tests for MDI or PDI, this study had a multiple-testing problem. If we performed the Bonferroni adjustment for addressing the multiple-testing problem, all the significances would have disappeared. Furthermore, this study has a small sample size. These are the limitations of this study.

However, because the measurement of PCBs and dioxins is highly complicated and is too expensive, it is difficult to measure these compounds in a large number of subjects. In recent studies of the associations between background-level exposure to PCBs and dioxins and infant neurodevelopment (Koopman-Esseboom et al. 1996; Walkowiak et al. 2001), the sample size was similar to our study. However, no studies have measured isomer levels of PCBs and dioxins to analyze the associations between individual isomer levels and infant neurodevelopment. Because exposure to PCBs and dioxins can be reduced to prevent their adverse effects on children’s development, even if the α error rate rises to some extent, we consider it important to catch these adverse effects on children’s development. Therefore, we believe that analysis of the associations between PCBs and dioxins and children’s development in this study was valuable.

In Japan, Nakai et al. (2004) also performed a prospective study to elucidate the influence of an endocrine-disrupting chemical on the neurodevelopment of children. Humans are the main source of various chemical compounds that are known pollutants, and food seems to be the main source of PCBs and dioxins in background-level exposure. Because the intake of marine products, such as fish or iodine, in an island country such as Japan would be greater than in western countries, a risk evaluation based on domestic dioxin/PCB data is important.

Vreugdenhil et al. (2002b) pointed out that an improving tendency was shown in children of school age, even if there was a significant negative association between endocrine-disrupting chemical level and neurodevelopment of children during infancy. Breast-feeding and a good home environment are regarded as important factors that improve the influence. Additional studies are needed to elucidate these results still further.

## Figures and Tables

**Table 1:** Characteristics of mothers and infants (*n* = 134).

Characteristic	No. (%)
Maternal characteristics	
Age (years)	31.1 ± 4.7^a^
Educational level (years)	
≤ 9	5 (3.7)
10–12	47 (35.1)
13–16	80 (59.7)
≥ 17	2 (1.5)
Economic status: annual income (yen)	
< 3,000,000	21 (15.7)
3,000,000–5,000,000	60 (44.8)
5,000,000–7,000,000	37 (27.6)
7,000,000–10,000,000	13 (9.7)
> 10,000,000	3 (2.2)
Worked during pregnancy	22 (16.4)
Smoked during pregnancy	18 (13.4)
Fish intake during pregnancy	
Inshore fish	
Rarely/never	8 (6.0)
< 1 time/week	67 (50.0)
1–4 times/week	58 (43.3)
≥ 5 times/week	1 (0.7)
Deep-sea fish	
Rarely/never	2 (1.5)
< 1 time/week	54 (40.3)
1–4 times/week	78 (58.2)
≥ 5 times/week	0
Caffeine intake during pregnancy (mg/day)	140.6 ± 91.5^a^
Alcohol intake before pregnancy (g/day)	19.7 ± 71.9^a^
Alcohol intake during pregnancy (g/day)	0.9 ± 2.6^a^
Blood sampling period	
During pregnancy	86 (64.2)
After delivery	48 (35.8)
Child characteristics	
Sex	
Male	68 (50.7)
Female	66 (49.3)
Gestational age (days)	277.3 ± 8.1^a^
Birth weight (g)	3124.8 ±329.1^a^
Length (cm)	48.2 ± 2.2^a^
Head circumference (cm)	33.4 ± 1.2^a^
First-born	67 (50.0)
Duration of breast-feeding, ≥ 3 months	78 (58.2)
Age at testing (days)	187.0 ± 4.4^a^
BSID-II mental index score: MDI	91.9 ± 5.8^a^
BSID-II motor index score: PDI	89.3 ± 10.5^a^
Index of Child Care Environment^b^	22.7 ± 2.4^a^

a Mean ± SD.

b A perfect score is 30 points.

**Table 2:** BSID-II mental (MDI) and psychomotor (PDI) development scores for infants in relation to mother and infant characteristics (*n* = 134).

		MDI		PDI	
Characteristic	No.	Mean ± SD	*p*-value	Mean ± SD	*p*-value
Maternal characteristics					
Age (years)		*r* = 0.042	0.626	*r* = −0.059	0.496
Educational level					
≤ 12 years	52	92.2 ± 5.0	0.647	89.8 ± 10.8	0.676
≥ 13 years	82	91.7 ± 6.2		89.0 ± 10.4	
Economic status: annual income					
< 5,000,000 yen	81	91.6 ± 5.8	0.444	89.4 ± 10.4	0.875
≥ 5,000,000 yen	53	92.3 ± 5.8		89.1 ± 10.7	
Worked during pregnancy					
No	112	91.5 ± 5.7	0.107	89.1 ± 10.3	0.697
Yes	22	93.7 ± 6.0		90.1 ± 11.8	
Smoked during pregnancy					
No	116	92.0 ± 5.8	0.469	89.8 ± 10.6	0.183
Yes	18	90.9 ± 5.8		86.2 ± 9.1	
Inshore fish intake during pregnancy					
< 1 time/week; rarely/never	75	91.6 ± 6.0	0.612	88.9 ± 11.3	0.670
≥ 1 time/week	59	92.2 ± 5.5		89.7 ± 9.4	
Deep-sea fish intake during pregnancy					
< 1 time/week; rarely/never	56	92.6 ± 5.3	0.198	89.9 ± 10.6	0.542
≥ 1 time/week	78	91.3 ± 6.0		88.8 ± 10.5	
Caffeine intake during pregnancy (mg/day)		*r* = −0.063	0.470	*r* = −0.177	0.040*
Alcohol intake before pregnancy (g/day)		*r* = −0.050	0.564	*r* = −0.099	0.256
Alcohol intake during pregnancy (g/day)		*r* = 0.137	0.114	*r* = 0.011	0.898
Blood sampling time					
During pregnancy	86	92.0 ± 5.5	0.767	88.8 ± 10.3	0.472
After delivery	48	91.7 ± 6.3		90.2 ± 10.9	
Child characteristics					
Sex					
Male	68	91.6 ± 5.8	0.595	87.8 ± 9.9	0.084
Female	66	92.1 ± 5.8		90.9 ± 10.9	
Gestational age (days)		*r* = 0.178	0.039*	*r* = 0.289	0.001**
Birth weight (g)		*r* = 0.103	0.237	*r* = 0.086	0.323
Length (cm)		*r* = 0.058	0.506	*r* = −0.060	0.489
Head circumference (cm)		*r* = 0.071	0.416	*r* = −0.015	0.862
First-born					
Yes	67	92.3 ± 6.1	0.356	89.7 ± 10.5	0.617
No	67	91.4 ± 5.5		88.8 ± 10.6	
Duration of breast-feeding, ≥ 3 months					
Yes	78	92.1 ± 6.0	0.557	89.9 ± 10.5	0.422
No	56	91.5 ± 5.5		88.4 ± 10.5	
Index of Child Care Environment		*r* = −0.016	0.855	*r* = −0.122	0.161

Student’s *t*-test, Pearson’s correlation coefficient test:

* *p* < 0.05;

** *p* < 0.01.

**Table 3:** Level of PCBs and dioxins (pg/g lipid) in maternal blood (*n* = 134).

	Detection limit^a^	Mean	Geometric mean	Minimum	25th^b^	50th^b^	75th^b^	Maximum
PCDDs								
2,3,7,8-TCDD	1.0	1.1	0.9	ND	ND	1.1	1.4	3.1
1,2,3,7,8-PeCDD	1.0	4.4	3.9	ND	3.1	4.2	5.4	11.9
1,2,3,4,7,8-HxCDD	2.0	1.8	1.6	ND	ND	ND	2.3	13.6
1,2,3,6,7,8-HxCDD	2.0	15.5	13.7	2.4	10.4	14.5	18.3	43.6
1,2,3,7,8,9-HxCDD	2.0	2.3	1.9	ND	ND	2.2	3.2	7.4
1,2,3,4,6,7,8-HpCDD	2.0	26.0	24.0	9.6	18.2	23.3	31.5	69.7
OCDD	4.0	504.7	468.0	169.6	352.2	467.4	601.2	1491.5
PCDFs								
2,3,7,8-TCDF	1.0	0.7	0.6	ND	ND	ND	ND	2.5
1,2,3,7,8-PeCDF	1.0	0.6	ND	ND	ND	ND	ND	2.2
2,3,4,7,8-PeCDF	1.0	6.5	5.8	1.4	4.5	6.0	7.7	19.9
1,2,3,4,7,8-HxCDF	2.0	2.6	2.3	ND	ND	2.6	3.3	6.5
1,2,3,6,7,8-HxCDF	2.0	3.0	2.6	ND	2.2	2.8	3.7	8.6
2,3,4,6,7,8-HxCDF	2.0	1.1	1.1	ND	ND	ND	ND	5.0
1,2,3,7,8,9-HxCDF	2.0	ND	ND	ND	ND	ND	ND	ND
1,2,3,4,6,7,8-HpCDF	2.0	3.1	2.4	ND	ND	2.5	3.4	15.8
1,2,3,4,7,8,9-HpCDF	2.0	ND	ND	ND	ND	ND	ND	ND
OCDF	4.0	2.1	ND	ND	ND	ND	ND	11.0
Non-*ortho* PCBs								
3,3′,4′,4′-TCB (77)	10.0	13.2	11.9	ND	10.5	12.8	16.1	37.2
3,4,4′,5-TCB (81)	10.0	ND	ND	ND	ND	ND	ND	ND
3,3′,4,4′,5-PeCB (126)	10.0	42.2	36.0	ND	26.0	37.0	54.9	141.7
3,3′,4,4′,5,5′-HxCB (169)	10.0	31.9	28.3	ND	21.5	29.2	36.6	85.9
Mono-*ortho* PCBs								
2,3,3′4,4′-PeCB (105)	10.0	1637.6	1418.8	256.1	976.2	1423.9	2044.1	5420.8
2,3,4,4′,5-PeCB (114)	10.0	407.2	353.3	79.3	248.6	363.9	488.5	1442.6
2,3′,4,4′,5-PeCB (118)	10.0	6582.0	5691.3	1293.2	3912.5	5803.5	8192.7	20196.9
2′,3,4,4′,5-PeCB (123)	10.0	127.8	109.4	24.3	70.3	113.7	154.5	458.6
2,3,3′,4,4′,5-HxCB (156)	10.0	2213.2	1942.1	441.5	1307.2	1982.9	2737.5	6427.8
2,3,3′,4,4′,5′-HxCB (157)	10.0	557.2	485.4	85.1	333.3	507.0	675.4	1782.7
2,3′,4,4′,5,5′-HxCB (167)	10.0	806.3	702.0	158.6	507.6	739.9	1005.0	2275.3
2,3,3′,4,4′,5,5′-HpCB (189)	10.0	244.7	209.2	ND	138.1	222.7	312.0	625.8
Di-*ortho* PCBs								
2,2′,3,3′,4,4′,5-HpCB (170)	10.0	4518.4	3881.1	1104.9	2570.7	4155.7	5622.0	13620.5
2,2′,3,4,4′,5,5′-HpCB (180)	10.0	13996.8	12063.7	2704.4	8392.9	12813.5	17957.1	41110.1
Total								
Total PCDDs		547.8	511.1	192.8	389.5	508.9	646.9	1602.4
Total PCDFs		42.7	21.1	10.4	16.6	20.6	24.5	2877.3
Total PCDDs/PCDFs		590.5	538.1	206.4	408.3	526.8	675.1	3726.3
Total non-*ortho* PCBs		91.4	83.7	27.4	64.2	85.5	110.3	269.9
Total mono-*ortho* PCBs		12575.9	11042.2	2832.8	7812.5	11471.6	15238.9	36382.2
Total coplanar PCBs		12667.3	11131.1	2860.2	7868.6	11554.7	15352.0	36536.1
Total		13257.8	11770.9	3311.1	8431.7	12053.1	15845.5	37267.2
WHO-98^c^								
Total PCDD TEQ		7.7	7.0	1.6	5.3	7.2	9.4	20.7
Total PCDF TEQ		4.2	3.8	1.2	2.9	3.8	5.0	12.4
Total PCDD/PCDF TEQ		11.9	10.9	2.8	8.2	11.2	14.0	31.2
Total non-*ortho* PCB TEQ		4.5	3.8	0.6	2.7	3.9	5.9	15.0
Total mono-*ortho* PCB TEQ		2.5	2.2	0.6	1.6	2.2	2.9	7.2
Total coplanar PCB TEQ		6.9	6.1	1.1	4.4	6.2	8.6	22.2
Total TEQ		18.8	17.2	4.0	13.4	17.8	23.4	51.2

Abbreviations: ND, nondetectable; WHO, World Health Organization.

a For subjects with a level below the detection limit, we used a value equal to half the detection limit.

b Percentiles.

c The calculation of TEQ was estimated based on the toxic equivalent factor values proposed by the WHO (Van den Berg 1998).

**Table 4:** BSID-II mental (MDI) and psychomotor (PDI) development scores for infants in relation to the level of PCBs and dioxins in maternal blood (*n* = 134).

	MDI			PDI		
	β^a^	*t*	*p*-Value	β^a^	*t*	*p*-Value
PCDDs						
2,3,7,8-TCDD	−0.154	−1.755	0.083	−0.125	−1.477	0.142
1,2,3,7,8-PeCDD	0.074	0.847	0.398	−0.063	−0.758	0.450
1,2,3,4,7,8-HxCDD	−0.031	−0.352	0.725	−0.135	−1.608	0.110
1,2,3,6,7,8-HxCDD	0.033	0.365	0.716	−0.074	−0.873	0.384
1,2,3,7,8,9-HxCDD	0.006	0.067	0.946	−0.209	−2.549	0.012*
1,2,3,4,6,7,8-HpCDD	−0.222	−2.418	0.017*	−0.243	−2.806	0.006**
OCDD	−0.177	−1.897	0.060	−0.168	−1.895	0.060
PCDFs						
2,3,7,8-TCDF	−0.053	−0.602	0.548	−0.204	−2.516	0.013*
1,2,3,7,8-PeCDF	0.014	0.167	0.868	−0.203	−2.512	0.013*
2,3,4,7,8-PeCDF	0.028	0.314	0.754	−0.069	−0.822	0.413
1,2,3,4,7,8-HxCDF	−0.113	−1.255	0.212	−0.153	−1.807	0.073
1,2,3,6,7,8-HxCDF	−0.101	−1.139	0.257	−0.166	−1.989	0.049*
2,3,4,6,7,8-HxCDF	0.009	0.106	0.915	−0.074	−0.875	0.383
1,2,3,7,8,9-HxCDF	ND	ND	ND	ND	ND	ND
1,2,3,4,6,7,8-HpCDF	−0.038	−0.438	0.662	−0.093	−1.123	0.264
1,2,3,4,7,8,9-HpCDF	ND	ND	ND	ND	ND	ND
OCDF	−0.057	−0.657	0.512	−0.032	−0.385	0.701
Non-*ortho* PCBs						
3,3′,4′,4′-TCB (77)	0.032	0.357	0.721	−0.023	−0.274	0.785
3,4,4′,5-TCB (81)	ND	ND	ND	ND	ND	ND
3,3′,4,4′,5-PeCB (126)	−0.003	−0.032	0.975	−0.138	−1.674	0.097
3,3′,4,4′,5,5′-HxCB (169)	0.013	0.147	0.884	−0.097	−1.181	0.240
Mono-*ortho* PCBs						
2,3,3′,4,4′-PeCB (105)	−0.004	−0.047	0.963	−0.112	−1.366	0.174
2,3,4,4′,5-PeCB (114)	−0.024	−0.277	0.782	−0.132	−1.607	0.111
2,3′,4,4′,5-PeCB (118)	−0.016	−0.182	0.856	−0.137	−1.671	0.097
2′,3,4,4′,5-PeCB (123)	0.033	0.379	0.705	−0.108	−1.307	0.194
2,3,3′,4,4′,5-HxCB (156)	0.001	0.008	0.994	−0.099	−1.201	0.232
2,3,3′,4,4′,5′-HxCB (157)	−0.038	−0.431	0.667	−0.141	−1.723	0.087
2,3′,4,4′,5,5′-HxCB (167)	−0.012	−0.140	0.889	−0.135	−1.652	0.101
2,3,3′,4,4′,5,5′-HpCB (189)	−0.102	−1.182	0.239	−0.136	−1.662	0.099
Di-*ortho* PCBs						
2,2′,3,3′,4,4′,5-HpCB (170)	−0.027	−0.310	0.757	−0.132	−1.612	0.109
2,2′,3,4,4′,5,5′-HpCB (180)	−0.021	−0.246	0.806	−0.086	−1.042	0.299
Total						
Total PCDDs	−0.234	−2.649	0.009**	−0.144	−1.686	0.094
Total PCDFs	−0.048	−0.535	0.594	0.071	0.833	0.407
Total PCDDs/PCDFs	−0.220	−2.485	0.014*	−0.090	−1.046	0.298
Total non-*ortho* PCBs	0.032	0.365	0.716	−0.006	−0.076	0.939
Total mono-*ortho* PCBs	−0.014	−0.165	0.869	−0.136	−1.663	0.099
Total coplanar PCBs	−0.013	−0.155	0.877	−0.135	−1.650	0.101
Total	−0.022	−0.247	0.805	−0.130	−1.584	0.116
WHO-98^b^						
Total PCDD TEQ	0.046	0.522	0.603	0.004	0.043	0.966
Total PCDF TEQ	0.016	0.182	0.856	0.057	0.671	0.503
Total PCDDs/PCDF TEQ	0.037	0.419	0.676	0.032	0.378	0.706
Total non-*ortho* PCB TEQ	0.020	0.233	0.816	−0.017	−0.204	0.838
Total mono-*ortho* PCB TEQ	−0.011	−0.123	0.902	−0.127	−1.544	0.125
Total coplanar PCB TEQ	0.032	0.371	0.711	−0.053	−0.636	0.526
Total TEQ	0.049	0.550	0.583	0.007	0.080	0.936

Abbreviations: ND, nondetectable; WHO, World Health Organization.

a β is the point increase in developmental score per PCB and dioxin level (natural logarithm) adjusted for gestational age (days), smoking during pregnancy, caffeine intake during pregnancy (mg/day), and blood sampling time.

b The calculation of TEQ was estimated based on the toxic equivalent factor values proposed by the WHO (Van den Berg 1998).

* *p* < 0.05;

** *p* < 0.01.
